# Modern Sociology

**Published:** 1901-10-12

**Authors:** 


### Modern Sociology.

### LEPER HOMES AND THE LESSONS TO BE LEARNT FROM THEM.

For long centuries leprosy had been prevalent in Europe. At last a time arrived when, with comparative rapidity, the disease faded away, so that, from that time forth, except in a few isolated districts, leprosy and all concerning it became a mere matter of history. Now leprosy is an infective disease, at least in this sense that its morbid anatomy is due to the growth of a micro-organism. Whatever the predisposing causes may be its essential and necessary *causa causans* is this bacillus, just as the essential cause of tubercle is the bacillus tuberculosis; and when we find that in the course of a few centuries the disease vanished from the face of a great continent, we naturally ask why this happened. The answer is important, for we cannot but believe that this comparatively rapid fading away of so grave a disease as leprosy from so large an area as the continent of Europe, over which it had held sway for a time longer than history tells us of, must have some important lessons in regard to the possible eradication of other diseases which, like leprosy, are of microbic origin. It seems to be the fact that about the time when leprosy began to diminish, a large number of leper houses had been established in various parts of Europe by benevolent persons under the influence of Christian teaching which urged men to good works, and by many it has been taken for granted that the extermination of the disease was largely due to the segregation of those affected with it, a segregation rendered possible by the existence of these leper houses. The deduction is obvious that if a disease of such a low personal infectivity as leprosy could be banished from Europe by such segregation as was afforded in these lazar houses, we ought to be able, and if able, we ought seriously to attempt to banish other diseases of microbic origin by aid of segregation and other means directly aimed at preventing the passage of germs from the affected to the healthy. It becomes very important then that we should carefully examine the premises from which this inference is drawn, and all the more so since Professor Koch has lately encouraged us to hope for the extermination of tuberculosis by pointing out what has been effected in the case of leprosy through the prevention of infection.

Mr. Jonathan Hutchinson discussing this subject says that if those who hope for the extermination of tuberculosis by preventing its spread by contagion have no firmer ground than this they are indeed building upon sand, and he asks, is it conceivable that in all these countries, distant from each other and diverse in custom, any measures of isolation such as were then practicable could have brought about the simultaneous and uninterrupted decline of such a disease as leprosy. He points out that many as were the leper houses there was no attempt to enforce universal segregation. Indeed for isolation as we understand it at the present day, these houses were practically useless. No doubt in all countries it was always the case that lepers, when they became disfigured by the disease, were shunned and regarded with loathing, and very probably in certain cases admission to the leper homes was accompanied by certain ceremonies in the course of which the outcast leper renounced the world and pledged himself to seek no intercourse with his fellows. But there are many sidelights which show that the isolation was by no means strictly carried out. It is clear that non-lepers were employed to take charge of the sick, that in some leper houses there were healthy inmates as well as sick ones; and that in some both the attendants and the patients had their wives with them. Moreover it is pretty plain that admission to the leper homes was a matter of favour, so much so that the punishment for breaking rules was mere expulsion. The leper houses were charitable asylums for people in sickness and distress, not a means for the protection of the healthy from infection. They were, moreover, chiefly provided for the inhabitants of towns, the country lepers being left mostly to their fate, which was to wander at large among the people. The proof is to be found in every page of the records of mediæval lazar houses. "In that of Troyes," says Mr. Hutchinson, "healthy women were employed as nurses, and it is on record that at a visitation in 1575 three of these were found to be pregnant, and in the case of one of them it was for the second time during her residence of 10 years. The same woman was accused of having brought prostitutes into the home. A male leper of the same home, who was convicted of immorality at a public-house, was so flogged and banished for five years." Evidently of isolation in the modern sense of the word there was none. "At many leper homes the punishment for breaches of discipline was simply expulsion, and the delinquent was sent to wander through the country.' Under such circumstances and in view of the long period during which the infection of leprosy, such as it is, must remain in operation, it is impossible to attribute to the leper homes any great influence in restraining the propagation of the infection or in causing that general decline of leprosy which ended in its almost universal cessation throughout the principal countries of Europe. Everything points to the presumption that the disappearance of leprosy was due to the removal of some widely acting predisposing cause, for, as Mr. Hutchinson says, not only was the decline of leprosy general and almost simultaneous, but after the decline there was no relapse, and although into most of the countries from which it had disappeared numerous cases of leprosy have since been imported, and for the most part no precautions observed, the disease has never shown any tendency to again become endemic. What this predisposing cause was is another question into which we need not enter. The point of importance is to get rid of the idea that the cessation of leprosy in Europe was due to the measures taken to prevent personal contagion, and not to allow ourselves to be caught by the attractive hypothesis that as segregation could put a stop to leprosy in the Middle Ages, the same proceeding, now taking the form of sanatoria for consumptives, will put a stop to the tuberculosis of to-day.

